# Common Threads: Aphidicolin-Inducible and Folate-Sensitive Fragile Sites in the Human Genome

**DOI:** 10.3389/fgene.2021.708860

**Published:** 2021-09-08

**Authors:** Rachel Adihe Lokanga, Daman Kumari, Karen Usdin

**Affiliations:** ^1^Cancer Genetics Branch, National Cancer Institute, Bethesda, MD, United States; ^2^Laboratory of Cell and Molecular Biology, National Institute of Diabetes, Digestive and Kidney Diseases, National Institutes of Health, Bethesda, MD, United States

**Keywords:** break-induced DNA replication, mitotic DNA synthesis, SLX1-SLX4, MUS81/EME1, replication fork blockage, R-loops, origins of replication, secondary DNA structures

## Abstract

The human genome has many chromosomal regions that are fragile, demonstrating chromatin breaks, gaps, or constrictions on exposure to replication stress. Common fragile sites (CFSs) are found widely distributed in the population, with the largest subset of these sites being induced by aphidicolin (APH). Other fragile sites are only found in a subset of the population. One group of these so-called rare fragile sites (RFSs) is induced by folate stress. APH-inducible CFSs are generally located in large transcriptionally active genes that are A + T rich and often enriched for tracts of AT-dinucleotide repeats. In contrast, all the folate-sensitive sites mapped to date consist of transcriptionally silenced CGG microsatellites. Thus, all the folate-sensitive fragile sites may have a very similar molecular basis that differs in key ways from that of the APH CFSs. The folate-sensitive FSs include FRAXA that is associated with Fragile X syndrome (FXS), the most common heritable form of intellectual disability. Both CFSs and RFSs can cause chromosomal abnormalities. Recent work suggests that both APH-inducible fragile sites and FRAXA undergo Mitotic DNA synthesis (MiDAS) when exposed to APH or folate stress, respectively. Interestingly, blocking MiDAS in both cases prevents chromosome fragility but increases the risk of chromosome mis-segregation. MiDAS of both APH-inducible and FRAXA involves conservative DNA replication and POLD3, an accessory subunit of the replicative polymerase Pol δ that is essential for break-induced replication (BIR). Thus, MiDAS is thought to proceed *via* some form of BIR-like process. This review will discuss the recent work that highlights the similarities and differences between these two groups of fragile sites and the growing evidence for the presence of many more novel fragile sites in the human genome.

## Introduction

Fragile sites are apparent as chromatin gaps, constrictions, or breaks in cells exposed to replication stress (Sutherland, 1991). These sites are typically classified based on the reagent that induces their expression most effectively. They are also classified as common or rare, depending on their frequency in the population (Feng and Chakraborty, 2017). The largest known group of fragile sites are most efficiently induced by aphidicolin (APH), an inhibitor of DNA polymerases α, δ, and ε. FRA3B and FRA16D are among the best known APH inducible CFSs. FRA3B is associated with the fragile histidine triad *(FHIT)* gene, a tumor suppressor gene located on chromosome 3p14.2 and FRA16D is associated with the WW domain-containing oxidoreductase *(WWOX)* gene, a tumor suppressor gene located on chromosome 16 (Bednarek et al., 2000). Another group of fragile sites are referred to as being folate-sensitive since they are induced by either too much or too little folate, with both situations resulting in nucleotide pool imbalances (Glover, 1981; James et al., 1993). Perhaps, the best known of the folate-sensitive fragile sites is the rare fragile site, FRAXA, a site on the X chromosome that is seen in individuals with fragile X syndrome (FXS), the most common heritable cause of intellectual disability and autism spectrum disorder (Lozano et al., 2014). Other fragile sites are induced by agents, such as 5-azacytidine, 5-bromo-2-deoxyuridine, or distamycin A that can be incorporated or intercalated into DNA (Schmid et al., 1980; Sutherland et al., 1985; Hori et al., 1988). Interestingly, FRA16B and FRA10B, two rare distamycin-inducible fragile sites, are AT-rich minisatellites (Yu et al., 1997; Hewett et al., 1998) that are expansions of the AT microsatellites normally present in the CFSs FRA16C and FRA10E, respectively (Zlotorynski et al., 2003). As such, they may share common features with the APH-inducible sites. While most fragile sites replicate late in the cell cycle, early replicating fragile sites (ERFSs) have also been identified that are readily induced by hydroxyurea, a reagent that causes depletion of deoxynucleotide pools (Barlow et al., 2013).

Fragile sites are all thought to be regions of the genome that for some reason are slow to complete replication, and their presence is associated with a variety of chromosome abnormalities. Genome instability at CFSs is thought to be a driving force for tumorigenesis with APH-CFSs being associated with copy number variations, including a variety of recurrent cancer deletions (Le Tallec et al., 2013; Wilson et al., 2015; Zheglo et al., 2019). Some CFS-associated CNVs are also associated with neurological disorders (Denison et al., 2003; Ambroziak et al., 2015; Zheglo et al., 2019). CFSs are also frequent sites of viral integration associated with cancer (Thorland et al., 2000; Yu et al., 2005). In contrast to the CNVs associated with CFSs, ERFSs are associated with recurrent chromosomal rearrangements during lymphomagenesis (Barlow et al., 2013). The RFS FRAXA is associated with a high frequency loss of the affected X chromosome *in vitro* in response to folate stress (Bjerregaard et al., 2018) and *in vivo* (Dobkin et al., 2009), and many cases of Jacobsen (11q-) syndrome, a chromosomal deletion disorder affecting chromosome 11, have been attributed to the presence of folate-sensitive fragile sites on that chromosome (Jones et al., 1994).

## The Molecular Basis of the Replication Problems At Cfss and Folate-Sensitive Fss

Unlike ERFSs which are located in early replicating G + C-rich, gene-dense regions with high numbers of activated origins of replication (ORIs) (Barlow et al., 2013), many APH-inducible CFSs are located in active, A + T-rich genes that are >300kb in size, replicate late, and are frequently ORI-poor (Glover et al., 2017; Debatisse and Rosselli, 2019). CFSs have been reported to be located at topologically associated domains (TADs) in some studies (Sarni et al., 2020), but not others (Ji et al., 2020). Some CFSs are associated with the expression of different oncogenes that can modulate replication stress (Miron et al., 2015). Transcription is required for CFS expression (Helmrich et al., 2011; Park et al., 2021), although higher transcription rates are associated with reduced fragility, perhaps due to the associated shift of the locus to replication earlier in the cell cycle (Blin et al., 2019). The relationship to transcription likely explains the reported tissue specificity of CFS expression.

Many different models have been proposed to account for the replication difficulties of CFSs, including those invoking replication-transcription collisions that promote R-loop formation and ultimately the stalling of the replication fork (Helmrich et al., 2011) and/or structural blocks to replication fork progression resulting from hairpin or cruciform formation by the AT-dinucleotide-rich regions embedded within many CFSs (Zlotorynski et al., 2003; Ozeri-Galai et al., 2011; Irony-Tur Sinai et al., 2019; Van Wietmarschen et al., 2020). In addition, TAD boundaries located between different replication timing zones are known to be prone to replication fork stalling (Lombardi and Tarsounas, 2020). Since ORIs are only licensed in G1 and bound pre-replication complexes can be displaced by RNA Pol II, at least in yeast (Snyder et al., 1988), it has also been suggested that transcription of long genes results in a paucity of active ORIs within the gene body that delays the completion of replication (Brison et al., 2019). Parenthetically, while a paucity of ORIs is associated with replication stress at CFSs, it has been suggested that increased ORI initiation at ERFSs also causes replication stress, perhaps by prematurely depleting nucleotide pools or by increasing replication-transcription collisions (Barlow et al., 2013).

However, while some studies support a role of R-loops in replication stress at fragile sites, including FRA3B (Helmrich et al., 2011), others do not (Park et al., 2021). Furthermore, while molecular combing has demonstrated replication stalling at FRA16C (Ozeri-Galai et al., 2011) and at FRA16D and FRA6E in *FANCD2*^−/−^ cells (Madireddy et al., 2016), combing studies of FRA3B and FRA6E showed no evidence of abnormal fork speed or replication fork stalling in normal APH-treated cells (Palumbo et al., 2010; Letessier et al., 2011). The lack of stalling at FRA3B together with the fact that transcription inhibition in S phase did not affect fragile site expression would be consistent with the idea that stalled replication forks and/or replication-transcription collisions are not a major source of replication stress at all CFSs (Brison et al., 2019). In addition, while delayed replication and their presence within large, transcriptionally active genes are consistent features of CFSs, these features are not sufficient for fragility, since a number of active, large genes that replicate late are not fragile (Wilson et al., 2015; Sarni et al., 2020; Park et al., 2021). Thus, the precise nature of the replication problem or problems at CFSs remains enigmatic and current thinking is that a combination of different factors may contribute to replication stress at different loci.

Unlike CFSs, many of the RFSs involve a much shorter region of DNA, usually 0.6–5kb. Of the 10 folate-sensitive RFSs characterized to date, all consist of a single tract of >200 CGG repeats (Table 1). In most cases, the repeat is in the 5' UTR of a gene that is epigenetically silenced (Lukusa and Fryns, 2008). Thus, fragility of these sites is likely to have a similar molecular basis. These sites are often associated with human disease, most commonly intellectual disability and autism spectrum disorder. However, it is not the fragile site itself that is responsible for this pathology, but rather the silencing-associated loss of the affected gene product. In the case of FRAXA and its associated disorder, FXS, the CGG repeat tract is located in the 5' UTR of the X-linked *FMR1* gene. The CGG repeat tract is prone to two forms of instability, the tendency to gain repeats with time, a hallmark of the repeat expansion diseases (Paulson, 2018) and the propensity to show fragility and sex chromosome aneuploidy (Dobkin et al., 2009). Both CGG repeats and the complementary CCG repeat form secondary structures, including hairpins and either G4 quadruplexes or i-motif structures [reviewed in Mirkin (2006)]. *In vitro* the CGG repeats show a K^+^ specific block to DNA synthesis consistent with the underlying problem being the formation of a G4 structure (Usdin and Woodford, 1995). The repeats also stall DNA synthesis in mammalian model systems (Voineagu et al., 2009) and in the endogenous *FMR1* locus (Gerhardt et al., 2014). In contrast to APH-inducible sites, the expression of FRAXA requires transcriptional silencing since those rare FXS alleles that escape silencing are not fragile (Yudkin et al., 2014). DNA methylation associated with gene silencing could increase the stability of fork blocking structures (Hardin et al., 1993; Lin et al., 2013). However, it is probable that silencing *per se* is not the trigger for fragility, but rather the delayed replication associated with silencing; transcribed *FMR1* alleles replicate late in the cell cycle, in S4 or G2 (Hansen et al., 2010), with silenced FXS alleles replicating even later (Webb, 1992; Hansen et al., 1997). Folate stress would delay this even further. FXS alleles lack the association with a TAD boundary that is seen in normal alleles (Sun et al., 2018). Thus, while APH CFSs and FRAXA share some common features, the underlying problems responsible for these different classes of fragile sites are likely to also be different.

**Table 1:** Folate-sensitive rare fragile sites known to be associated with CGG microsatellites.

Fragile site/disorder	Gene	References
FRA2A ID	*AFF3*	Metsu et al., 2014b
FRA7A autism spectrum disorder	*ZNF713*	Metsu et al., 2014a
FRA10A^*^	*FRA10AC1*	Sarafidou et al., 2004
FRA11A^*^	*C11orf80*	Debacker et al., 2007
FRA11B^§^	*CBL2*	Jones et al., 1994
FRA12A ID	*DIP2B*	Winnepenninckx et al., 2007
FRA16A^*^	*XYLT1*	Nancarrow et al., 1994
FRAXA ID/*FMR1* disorders	*FMR1*	Verkerk et al., 1991
FRAXE ID	*FMR2/AFF2*	Knight et al., 1993
FRAXF^*^	*FAM11A*	Parrish et al., 1994; Shaw et al., 2002

* *not associated with disease.*

§ *responsible for some cases of Jacobsen syndrome, a chromosome deletion syndrome.*

## The Downstream Consequences of the Replication Problems At Fragile Sites

Both APH-inducible CFSs and the FRAXA locus undergo mitotic DNA synthesis (MiDAS; Minocherhomji et al., 2015; Bhowmick et al., 2016; Garribba et al., 2020), a salvage pathway that ensures that regions of the genome that have not completed replication by the start of mitosis are successfully duplicated before the cell divides (Minocherhomji et al., 2015). Given that folate-sensitive fragile sites are all comprised of long CGG microsatellites, it is reasonable to think that other folate-sensitive fragile sites undergo MiDAS as well. MiDAS at both APH-inducible CFSs and FRAXA shares some common features. Both proceed *via* conservative DNA replication, in which DNA synthesis is confined to just one of the sister chromatids. They both also require POLD3 (Bhowmick et al., 2016; Garribba et al., 2020). POLD3, an accessory subunit of the replicative polymerase Pol δ, is not required for normal chromosomal replication, but is required for break-induced replication (BIR) (Costantino et al., 2014), a form of homologous recombination involved in the repair of one-sided double-strand breaks (DSBs) arising at collapsed replication forks. Thus, MiDAS has many of the hallmarks of a BIR-related process. BIR usually proceeds *via* the cleavage of the leading strand template by one of the structure-selective endonucleases: XPF-ERCC1, MUS81-EME1, or SLX1-SLX4. Cleavage results in a free 3' DNA tail that can strand-invade the sister chromatid to create a D-loop thus allowing POLD3-dependent DNA synthesis to proceed using the sister chromatid as a template. Successful completion of BIR at fragile site loci results in completely replicated chromatids that can be properly segregated into daughter cells in anaphase. Inhibition of BIR, on the other hand, results in the reduced expression of both CFSs and FRAXA, consistent with the idea that fragility is an active process resulting from MiDAS that has not been completed by the time normal chromatin condensation begins (Minocherhomji et al., 2015; Garribba et al., 2020). BIR frequently involves multiple rounds of strand invasion, DNA synthesis, and dissociation (Smith et al., 2007). Dissociation at one interspersed repeat and reinvasion into a different one could produce deletions, if the second repeat was downstream of the first one, and duplications if upstream. This could contribute to the high incidence of CNVs associated with fragile sites. Repeated mispriming within the repeat tract during BIR could also account for the tendency of CGG repeat tracts to expand (Kononenko et al., 2018). However, in the case of CGG repeats at the *FMR1* locus expansions occur in cells like ova that do not replicate (Yrigollen et al., 2014; Zhao and Usdin, 2018) and, in contrast to fragility, expansion at this locus requires transcription (Lokanga et al., 2014). Thus, the trigger for fragility and expansion of CGG repeats may differ.

**Figure 1:**
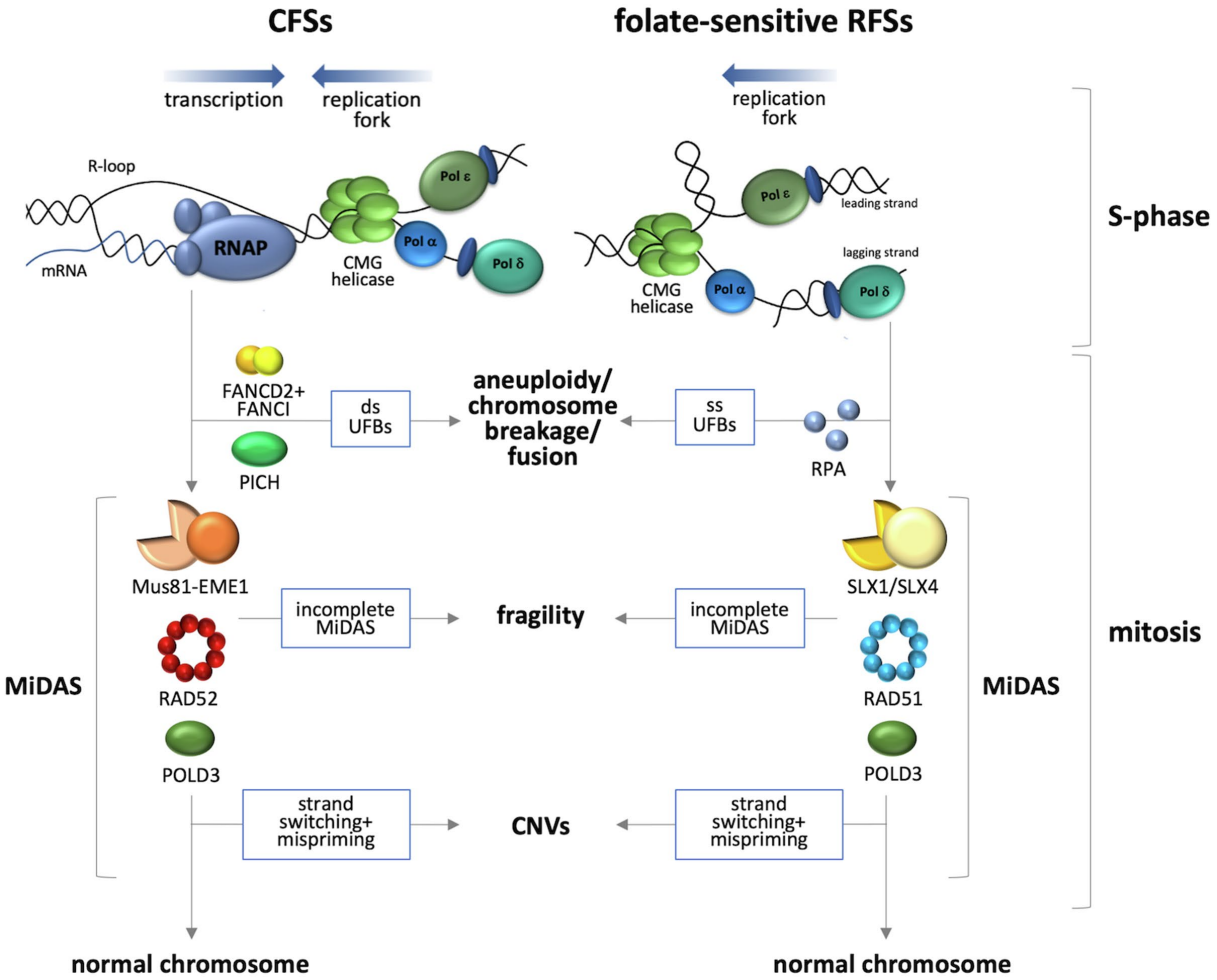
Current models for events occurring at loci containing APH-inducible and folate-sensitive FSs. APH-inducible sites are difficult to replicate, a situation that may be exacerbated by collisions between the replication fork and the transcription complex. A head-on collision is depicted here since it is associated with elevated levels of DSBs as well as the formation of R-loops (Hamperl et al., 2017) suggested to be important for fragility of these sites (Helmrich et al., 2011). Folate-sensitive RFSs are associated with replication problems resulting from the formation of a fork blocking lesion at a locus that is transcriptionally silent. Rescue of the stalled replication forks occurs by MiDAS that involves two different BIR subpathways (Wyatt et al., 2013; Ying et al., 2013; Minocherhomji et al., 2015; Bhowmick et al., 2016; Garribba et al., 2020). Completion of MiDAS produces a normal chromosome, while failure to do so results in chromosome fragility. Strand switching during BIR that results in mispriming can result in CNVs. Failure to initiate MiDAS results in either double-stranded UFBs in the case of the APH sites (Chan et al., 2007) or single-stranded UFBs in the case of FRAXA (Bjerregaard et al., 2018).

While both the expression of CFSs and FRAXA likely involve some form of BIR, the process at these sites differs with respect to some of the proteins involved as illustrated in Figure 1. Specifically, initiation of BIR at APH-inducible sites involves cleavage of the replication intermediates by Mus81-EME1 (Ying et al., 2013) acting in conjunction with the scaffolding protein, SLX4 (Minocherhomji et al., 2015). Processing of the cleavage product requires Rad52 (Ying et al., 2013). In contrast, BIR at FRAXA requires the RAD51 recombinase and the SLX1-SLX4 endonuclease (Garribba et al., 2020). It has been suggested that the DNA secondary structures formed by the CGG repeat tract result in an atypical stalled fork that is a poor substrate for MUS81-EME1 (Garribba et al., 2020), a complex that specifically nicks duplex DNA on the 5'-side of a single-stranded/double-stranded DNA branch point (Wyatt et al., 2013). In contrast, the SLX1 endonuclease, which is activated by binding to SLX4, has a wider range of possible substrates and can incise duplex or single-stranded DNA on either the 5'- or 3'-sides of the branch point, thus allowing SLX1-SLX4 to nick either the leading or lagging strand template (Wyatt et al., 2013). This difference in substrates may account for the involvement of different enzymes for processing stalled replication forks in the case of CFSs and FRAXA (Garribba et al., 2020). Interestingly, in a tissue culture reporter system, siRNA knockdown of either RAD51 or RAD52 significantly reduced BIR-associated mutagenesis of the region flanking a CGG repeat tract (Kononenko et al., 2018). Whether this reflects two different BIR subpathways operating in these cells or some sort of hybrid process is unclear.

While initiation of MiDAS is required for cytogenetic expression of the fragile site, failure to initiate MiDAS at both sets of loci leads to increased formation of ultrafine bridges (UFBs) in anaphase (Minocherhomji et al., 2015; Bhowmick et al., 2016; Garribba et al., 2020). These UFBs are anaphase bridges that do not stain with conventional DNA stains like Hoechst or DAPI and are not associated with histones (Chan et al., 2009). Failure to resolve these UFBs results the formation of micronuclei and chromosome mis-segregation (Fernandez-Casanas and Chan, 2018). Perhaps not surprisingly given the differences in the underlying cause of replication fork stalling, CFSs and the FRAXA locus also differ in the nature of the UFBs that are formed when MiDAS does not occur. The UFBs formed at APH-inducible sites are coated with PICH, a DNA translocase, are double-stranded (Liu et al., 2014), and are bounded by FANCD2/FANCI foci (Chan et al., 2009). The absence of an effect of topoisomerase II inhibition on the frequency of these UFBs suggests that they reflect the presence of under-replicated DNA or unresolved replication intermediates rather than dsDNA catenanes (Chan et al., 2009). The UFBs associated with FRAXA on the other hand are not associated with FANCD2, FANCI, or PICH. Instead, they are coated with RPA (Bjerregaard et al., 2018) and are thus likely to be single-stranded, consistent with unresolved HR intermediates (Fernandez-Casanas and Chan, 2018).

## Concluding Remarks

Lessons learnt from these two groups of fragile sites have allowed many more potential fragile sites to be identified. For example, genome-wide mapping of loci that undergo MiDAS in the presence of APH has identified hundreds of potential new CFSs (Ji et al., 2020; Macheret et al., 2020). Genome-wide studies of loci showing a fragility signature consisting of a TAD boundary that overlaps a highly transcribed, large gene with APH-induced replication delay, also suggest the presence of additional sites (Sarni et al., 2020). In addition, folate stress induces MiDAS or γ-H2AX foci, a marker of DSBs, at many genomic loci in normal human cells (Kumari et al., 2009; Garribba et al., 2020), suggesting that there are also several common folate-sensitive fragile sites that are as yet uncharacterized. Furthermore, a recent study of epigenetic variation in the human genome suggests the existence of at least 19 rare, long, and silenced CGG repeat tracts that could well also be fragile (Garg et al., 2020).

In addition to the CGG repeat diseases associated with folate-sensitive RFSs, many other repeat expansion diseases are known (Paulson, 2018). The CTG repeats responsible for a subset of these disorders, block replication (Samadashwily et al., 1997; Pelletier et al., 2003), induce BIR (Kim et al., 2017), and cause chromosome fragility in yeast (Freudenreich et al., 1998; Freudenreich and Lahiri, 2004). They also block replication in human cells (Liu et al., 2012). Furthermore, when cells containing a reporter construct with (CTG)_100_ repeats were treated with hydroxyurea replication-dependent DSBs were seen close to the replication fork (Gadgil et al., 2020). Increased fragility as evidenced by the loss of an adjacent fluorescent reporter was also seen along with evidence of BIR. Interestingly, unlike BIR at the FRAXA locus, this BIR was dependent on MUS81 (Gadgil et al., 2020). CTG and CAG repeats form hairpins (reviewed in Mirkin, 2006), like the CGG and CCG repeats responsible for FRAXA. However, they do not form G4 or i-motif structures. The MUS81 requirement for fragility may reflect this difference. Since some of the CTG/CAG expansion disorders can involve thousands of repeats (Fu et al., 1992; Mahadevan et al., 1992; Van Kuilenburg et al., 2019), they may also be fragile. However, no mitotic fragility has been reported for individuals with one such disorder, myotonic dystrophy type 1 (DM1; Jalal et al., 1993; Wenger et al., 1996). This may reflect the fact that, according to the ENCODE dataset, DMPK, the affected gene, replicates in the G1 or G1b phase of the cell cycle (Thurman et al., 2007; Hansen et al., 2010). Similarly, the GAA repeat tract responsible for Friedreich ataxia has key hallmarks of a mammalian fragile site: It blocks DNA synthesis (Krasilnikova and Mirkin, 2004; Gerhardt et al., 2016; Murat et al., 2020), is fragile in yeast (Kim et al., 2008), and is prone to chromosomal duplications in culture (Kumari et al., 2015). It is also associated with a high frequency of *de novo* mutations in the flanking regions (Bidichandani et al., 1999), a hallmark of BIR. However, as with the DM1 repeats, these loci are not apparent as gaps or constrictions in the chromatin in metaphase spreads, perhaps because they too replicate early in S phase (Kumari et al., 2015).

Thus, the repeats responsible for the repeat expansion diseases, may represent an unappreciated double threat to the human genome: the first threat being mediated *via* the deleterious effects of having large numbers of repeats in the DNA, RNA, and/or protein encoded by the affected loci (Paulson, 2018) and the second posed by the difficulty of replicating the repeats, with downstream effects on genome integrity, including aneuploidy, translocations, and CNVs. In addition to the repeats currently known to be associated with pathology, many thousands of other microsatellites with potential to stall DNA replication are known to be present in the human genome (Murat et al., 2020). Thus, the number of potentially fragile sites in the human genome could well be much higher than currently appreciated.

## Author Contributions

All authors contributed equally to this manuscript.

## Funding

Funding for this work is provided by a grant from the Intramural Program of NIDDK, NIH to KU (1ZIADK057808).

## Conflict of Interest

The authors declare that the research was conducted in the absence of any commercial or financial relationships that could be construed as a potential conflict of interest.

## Publisher’s Note

All claims expressed in this article are solely those of the authors and do not necessarily represent those of their affiliated organizations, or those of the publisher, the editors and the reviewers. Any product that may be evaluated in this article, or claim that may be made by its manufacturer, is not guaranteed or endorsed by the publisher.
